# Serum sclerostin is an independent predictor of mortality in hemodialysis patients

**DOI:** 10.1186/1471-2369-15-190

**Published:** 2014-12-02

**Authors:** Flávia Letícia Carvalho Gonçalves, Rosilene M Elias, Luciene M dos Reis, Fabiana G Graciolli, Fernando Godinho Zampieri, Rodrigo B Oliveira, Vanda Jorgetti, Rosa MA Moysés

**Affiliations:** Nephrology Division, Universidade de São Paulo, São Paulo, Brazil; Emergency Medicine Division, Universidade de São Paulo, São Paulo, Brazil; Universidade Nove de Julho (UNINOVE), Rua Iperoig, 690 ap 121 – Perdizes, São Paulo, 05016-000 SP Brazil

**Keywords:** Sclerostin, Chronic renal failure, CKD-MBD, Hemodialysis, Mortality risk

## Abstract

**Background:**

Sclerostin (Scl) has recently emerged as a novel marker of bone remodeling and vascular calcification. However, whether high circulating Scl is also a risk factor for death is not well established. The purpose of this study was to test whether serum Scl would be associated with mortality.

**Methods:**

we measured serum Scl in a hemodialysis patients’ cohort, which was followed during a ten-year period. Competing risk regression models were applied, as during the follow-up, patients were exposed to both events kidney transplant and death.

**Results:**

Ninety-one patients aged 42.3 ± 18.8 years (55% of male gender, 15% of diabetes) were included. During the follow-up, 32 patients underwent kidney transplant and 26 patients died. Non-survivals presented higher FGF23, higher Scl and lower creatinine. There was an association between all-cause mortality and higher Scl (HR = 2.2), higher age (HR = 1.04) and presence of diabetes (HR = 2.27), by competing risk analyses. Even including potential markers of mortality, as creatinine, FGF 23, and gender, Scl, age and diabetes remained significantly related to higher mortality.

**Conclusion:**

Serum Scl is an independent predictor of mortality in dialysis patients. However, whether clinical interventions to modulate Scl would be able to improve these patients survival needs to be determined.

## Background

Chronic kidney disease-mineral and bone disorder (CKD-MBD) is a major syndrome that develops as a complication of CKD [1] and usually predicts outcomes in observational studies [2, 3]. However, the exact mechanisms that could explain the link between CKD-MBD and a higher mortality risk have not been completely unveiled. Increasing our challenge, recent studies have identified new molecules synthetized by the osteocytes, such as fibroblast growth factor 23 (FGF 23), which works as a powerful predictor of mortality and cardiovascular events in CKD [4]. In accordance to this, the osteocytes have been recognized as major cells that orchestrate bone remodeling through several molecular mechanisms [5] and recent studies are trying to elucidate the role of these cells in CKD-MBD pathophysiology [6].

The glycoprotein sclerostin (Scl) is synthetized by the osteocytes, and inhibits the bone anabolic Wnt pathway, which promotes bone formation [5]. It has already been shown that serum levels of Scl are elevated in hemodialysis patients, with an inverse correlation to serum parathormone (PTH) and bone formation rate [7]. In addition, we have recently described that increased bone expression of Scl is a common and early event in these patients [8], and probably plays a role in CKD-MBD pathophysiology, potentially increasing bone resistance to PTH. As a consequence, we would expect that higher Scl would be associated with worse outcomes. Surprisingly, it has recently shown in hemodialysis patients that high circulating Scl levels are associated with improved survival instead [9].

Therefore, there is still some debate on whether circulating Scl could be a new CKD-MBD marker of outcome. In addition, the potential relationship of serum Scl with another CKD-MBD markers, such as FGF 23, serum soluble Klotho (sKlotho) or recently described Scl modulators, such as inflammation, calcitonin [10] and others, have not yet been evaluated.

Trying to address these questions, we tested whether serum Scl would be associated with mortality in a hemodialysis patients’ cohort, which was followed during a ten-year period.

## Methods

### Study population

This study was done in a cohort of all prevalent patients who underwent chronic hemodialysis treatment in Hospital das Clinicas, Universidade de São Paulo, Brazil on January of 2003 (N = 91). All patients were on high-flux, high efficiency hemodialysis, blood flow of 350 ml/min, dialysate flow of 800 ml/min, with bicarbonate buffer and a polysulfone dialyzer. The schedule was 3.5 to 4.0 hours thrice a week. The dialysate content of calcium was 1.75 mmol/l. Clinical and biochemical data were collected and serum samples were stored for further analysis on January 2003. This study protocol was reviewed and approved by the Institutional Research Ethics Committee (Comissão de Ética para Análise de Projetos de Pesquisa do HCFMUSP; CAPPesq # 235.350). Written consent was obtained from each patient.

### Demographic, clinical, and laboratory data

Data on age, sex, diabetes mellitus, history of cardiovascular disease, and dialysis vintage were collected when the patients entered the study. Laboratory values of hemoglobin, creatinine, albumin, ferritin, calcium, phosphate, alkaline phosphatase [reference range (RR) = 32–122 U/L], parathyroid hormone (RR = 11–62 pg/ml, immunoradiometric assay) and β_2_ microglobulin (RR = 1.0–1.7 μg/ml, chemiluminescent assay) were collected on January 2003. On the same day, blood samples were collected and frozen serum samples were stored for further analyses. We measured calcitonin (RR for men < 8.4 pg/ml; women < 5 pg/ml; immunochemiluminometric assay), interleukin-6 (IL-6; ELISA assay; RR = ND-12.5 pg/ml; R&D Systems, MN, USA). sKlotho (ELISA assay, RR = 239 to 1266 pg/ml; IBL America, MN, USA); FGF 23 (cterm FGF 23, ELISA assay; RR = 55 ± 50 RU/ml; Immutopics, CA, USA), and Scl manufactured by Teco (ELISA assay; reference range: 0.56 ± 0.13 ng/ml for premenopausal women; 0.69 ± 0.20 ng/ml for postmenopausal women and 0.74 ± 0.27 ng/ml for men; intra- and interassay coefficients of variation = 3.1 and 3.5%, respectively; Sissach, Switzerland).

### Survival analyses

Data on all-cause death were obtained from local electronic and paper records. Patients were followed for survival for up to 10 years. Patients were censored if they underwent renal transplantation (n = 32) or ended the study, which ever happened first. During this follow-up, 26 patients died and causes of death were collected from medical records, defined as cardiovascular or non-cardiovascular.

### Statistical analyses

Categorical variables are presented as percentages and continuous variables as mean ± SD or median (25, 75 interquartile), where appropriate. A chi-squared test was used to evaluate differences in numbers of deaths between Scl higher and lower than median limits. Differences between groups were checked with ANOVA, t-test or Kruskal Wallis and Mann Whitney depended on normal or skewed distribution, respectively. Spearman correlation was done between Scl and other variables. Since renal transplantation was a competitor for mortality on follow-up in our study, a competing risk regression was used. Competing risk regression was performed using the approach suggested by Fine and Gray [11–14] Variables elected for the regression were those associated with mortality on univariate analysis and those defined *a priori* of clinical relevance (such as age and gender). This first model built was considered the initial model of this analysis. A second model including Scl levels stratified in high (above mean values) and low (bellow mean values) was also planned for graphical representation. We subsequently explored the variables included in the initial model by adding one variable at a time to a new model and evaluated the behavior of the Bayesian information criteria (BIC) of all models created in this way. The model with the smallest BIC value was defined as the final model [13]. The statistical analysis was done using *GraphPad Prism® software* (GraphPad Software, Inc., CA, USA) and R (version 3.0.3, “Warm Puppy” – http://www.r-project.org) with the following packages: *survival* and *cmprsk*. Two-tailed P value < 0.05 was considered statistically significant.

## Results

### Population characteristics and events

Baseline clinical and demographic characteristics of the study population are presented in Table 1. Our patients were relatively young and with a low percentage of diabetes. Despite that, serum Scl measured was elevated. We also observed that serum FGF 23, as well as IL-6 were higher than normal, whereas low sKlotho was found. During the follow-up period, 32 patients were submitted to kidney transplant, and 5 patients were transferred to other dialysis centers. There were 26 deaths. The main cause of death was cardiovascular (CV; 16 patients; due to myocardial infarction, stroke and aortic aneurysms), followed by infection (6 cases). Non-survivors were older and more frequently had diabetes. They also had higher mean values of FGF 23 and Scl, and a lower mean value for serum creatinine and sKlotho, as shown in Table 2. When we compared those patients who died from CV causes to survivors, we observed that they also were older and with a high percentage of diabetes, with higher FGF 23 and Scl and lower serum creatinine and sKlotho. The comparison of patients who died from other causes to survivors revealed that they presented lower hemoglobin and creatinine and higher IL-6. Finally, patients who died from CV causes were older than those who died from other causes.

**Table 1:** **Baseline characteristics (N = 91)**

		RR
Age, years	42.3 ± 18.8	-
Gender male/female	55/36	-
Dialysis vintage, months	120 (48, 168)	-
Diabetes, n (%)	14 (15.4)	-
Hemoglobin, g/dl	11.5 ± 1.6	13-18
Creatinine, mg/dl	11.0 ± 3.3	0.7-1.2
Albumin, g/dl	4.2 ± 0.4	3.4-4.8
Ferritin, ng/ml	523 (198, 814)	30-400
Calcium, mg/dl	9.2 ± 1.3	8.6-10.2
Phosphate, mg/dl	5.2 ± 1.9	2.7-4.5
Alkaline phosphatase, U/l	82.5 (64.7, 121.8)	32-122
Parathormone, pg/ml	169 (94, 325)	11-62
Calcitonin, pg/ml	2.8 (2.0, 5.0)	M < 8.4; F < 5
β_2_ microglobulin, μg/ml	27 (22, 34)	1.0-1.7
IL-6, pg/ml	3.0 (1.7, 5.0)	ND-12.5
sKlotho, pg/ml	40.6 (2.8, 83.6)	239-1266
FGF23, RU/ml	135.3 (125.8, 166.9)	5-105
Scl, ng/ml	0.88 (0.54, 1.55)	M: 0.5-1.0; F: 0.5-0.9

RR: Reference Range; M: male; F: female; AP: Alkaline Phosphatase; PTH: parathormone; Scl: sclerostin.

**Table 2:** **Survivors and non-survivors**

	Survivors (n = 65)	Non-survivors (n = 26)		
		All (CV +other causes)	CV cause (n = 16)	Other causes (n = 10)
Age, years	37.0 ± 13.6	55.4 ± 23.2^a^	63.0 ± 20.6^a^	43.3 ± 22.8^c^
Gender M/F	37/28	18/8	11/5	7/3
Dialysis vintage, mo	132 (48, 180)	84 (63, 105)	96 (48, 126)	72 (60,84)
Diabetes, n	5	9^a^	7^a^	2
Hemoglobin, g/dl	11.7 ± 1.5	11.0 ± 1.7	11.4 ± 1.7	10.6 ± 1.6^a^
Creatinine, mg/dl	11.6 ± 3.3	9.5 ± 2.9^a^	9.8 ± 2.6^a^	9.0 ± 3.5^a^
Albumin, g/dl	4.2 ± 0.4	4.1 ± 0.6	4.1 ± 0.7	4.1 ± 0.5
Ferritin, ng/ml	519 (175, 691)	570 (278, 902)	598 (270, 862)	570 (276,1106)
Calcium, mg/dl	9.1 ± 1.0	9.4 ± 1.3	9.5 ± 1.6	9.4 ± 0.9
Phosphate, mg/dl	5.4 ± 1.9	4.8 ± 1.8	5.3 ± 1.9	3.9 ± 1.3^a^
AP, U/l	82 (65, 122)	81 (68, 127)	77 (63, 107)	96 (68,158)
PTH, pg/ml	200 (97, 401)	133 (77, 229)	145 (91, 454)	132 (39,180)
Calcitonin, pg/ml	2.8 (2.0,4.9)	3.2 (2.0, 5.7)	2.0 (2.0, 4.82)	4.6 (2.0, 7.6)
β_2_micro, μg/ml	28 (22, 34)	25 (16, 30)	24 (19, 30)	30 (12, 45)
IL-6, pg/ml	2.8 (1.4, 4.2)	3.3 (2.3, 12.7)	3.0 (2.3, 6.9)	12.7 (2.0, 27.9)^a^
Klotho, pg/ml	46.8 (7.6, 82.8)	15.7 (0.1, 103.0)	8.7 (0.1, 45.1)^b^	114.3 (3.7, 167.7)
FGF 23, RU/ml	134 (122, 159)	147 (129, 197)^a^	150 (129,311)^a^	143 (133, 152)
Scl, ng/ml	0.76 (0.50, 1.34)	1.45 (0.83, 1.79)^a^	1.63 (1.20, 1.87)^a^	0.84 (0.45, 1.73)

M: male; F: female; AP: Alkaline Phosphatase; PTH: parathormone; β_2_micro: β_2_microglobulin; IL-6: interleucin; FGF 23: fibroblast growth factor; Scl: sclerostin; ^a^p < 0.05 vs. survivors; ^b^p = 0.052 vs. survivors; ^c^p < 0.05 vs. CV cause.

Serum Scl was higher in men than women [1.29 ng/ml (0.71, 1.65) vs. 0.71 ng/ml (0.42, 1.02); p = 0.001]. There was no difference in Scl levels regarding diabetes and age.

Subsequently, patients were divided according to Scl high or low (Table 3). Patients with high Scl were on dialysis for longer, had higher β_2_ microglobulin, and higher FGF 23. There was a tendency toward to present a higher serum calcitonin. Age, sex and diabetes were not different. Most cardiovascular and all-cause mortality were seen in the high Scl group.

**Table 3:** **Comparison between patients with low and high Sclerostin**

Variable	Low Scl (< 0.88 ng/ml) N = 45	High Scl (≥ 0.88 ng/ml) N = 46	p
Calcitonin, pg/ml	2.3 (2.0, 3.4)	3.5 (2.0, 6.0)	0.082
Age, years	42 ± 18	43 ± 20	0.966
Gender M/F	24/22	31/14	0.103
Diabetes, n	7	7	0.964
Dialysis vintage, mos	84 (36, 144)	144 (75, 180)	0.049
Creatinine, mg/dl	10.7 ± 3.6	11.3 ± 3.1	0.372
AP, U/l	106.9 ± 93.2	108.8 ± 72.6	0.984
Calcium, mg/dl	9.2 ± 1.0	9.2 ± 1.2	0.707
Phosphate, mg/dl	5.3 ± 1.7	5.2 ± 2.1	0.877
Hemoglobin, g/dl	11.7 ± 1.3	11.4 ± 1.8	0.468
PTH, pg/ml	133 (92, 363)	204 (91, 297)	0.835
Albumin, g/dl	4.1 ± 0.4	4.2 ± 0.5	0.224
Ferritin, ng/ml	558 (203, 851)	510 (196, 717)	0.685
β_2_micro, μg/ml	25.3 ± 9.6	30.0 ± 10.4	0.016
IL-6, pg/ml	2.6 (1.6, 3.8)	3.2 (1.9, 6.7)	0.112
Klotho, pg/ml	40.5 (4.6, 83.3)	40.8 (0.1, 84.6)	0.852
FGF 23, RU/ml	134 (123, 145)	149 (127, 212)	0.007
All-Cause mortality, n	8	18	0.012
CV mortality, n	1	15	0.0001

AP: Alkaline Phosphatase; PTH: parathormone; β_2_micro: β_2_microglobulin; IL-6: interleucin; FGF 23, fibroblast growth factor; CV: cardiovascular.

There was a relationship between Scl and calcitonin (r = 0.224, p = 0.035), β_2_ microglobulin (r = 0.333, p = 0.001), IL-6 (r = 0.251, p = 0.027), and FGF 23 (r = 0.331, p = 0.002). There was no correlation between Scl and PTH or between Scl and age.

**Figure 1:**
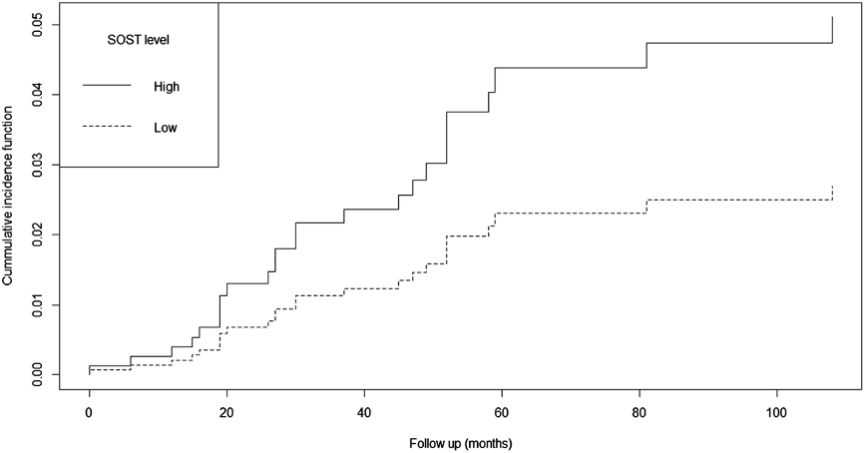
**Cumulative incidence function for Scl levels during follow up.** The regression included age, gender, Scl levels stratified in two categories (high versus low), FGF23 levels and presence of diabetes.

Age, creatinine, Scl levels, FGF23 levels, gender and presence of diabetes were elected for competing risk regression. The outcome of interest was defined as mortality, with transplantation as competitor. Results for this initial model are shown in Table 4 (top frame). Scl levels were positively associated with increased mortality over the follow-up period (HR 2.18; 95% CI 1.41-3.38). When Scl levels were categorized as high and low while keeping all other variables, high Scl levels were associated with a HR of 2.88 (95% CI 1.35-6.15). Cumulative incidence function for high Scl levels for mortality is shown in Figure 1. After the additive model exploration, the final model (with lowest BIC values) included only, age (HR 1.04; 95% CI 1.02-1.07), Scl levels (HR 2.20; 95% CI 1.35-3.56) and presence of diabetes (HR 2.27; 95% CI 1.14-4.54), as shown in Table 1 (bottom frame). Due to limited sample size, dialysis vintage was modeled in a distinct model, confirming that high Scl was still associated with mortality with an Exp (coef) of 2.98; 95% CI 1.35-6.33. The other variables retained were also age and diabetes.

**Table 4:** **Results for competing risk regression**

Initial model			
Variable	Exp (coef)	95% confidence interval	p
Age (per year)	1.03	1.00-1.06	0.012
Creatinine (per unit)	0.86	0.74-1.01	0.059
Scl (per unit)	2.18	1.41-3.38	<0.001
FGF23 (per unit)	1.00	0.99-1.00	0.400
Male sex (versus female)	0.82	0.39-1.75	0.620
Diabetes	2.40	1.16-4.98	0.018
**Final model**			
**Variable**	**Exp (coef)**	**95% confidence interval**	**p**
Age (per year)	1.04	1.02-1.07	<0.001
Scl (per unit)	2.20	1.35-3.56	0.001
Diabetes	2.27	1.14-4.54	0.020

Scl, sclerostin, FGF23, fibroblast grow factor 23.

## Discussion

The major finding of the present study was that high Scl was associated with CV mortality in prevalent dialysis patients, independent of diabetes and age, in a competing analysis approach.

In the last 20 years, several studies have tried to elucidate the physiopathology of CKD-MBD and the link between some of the components of this syndrome and the high mortality usually seen in CKD patients [15]. Concomitantly, the better understanding of the role of the Wnt pathway and its inhibitors has shed light on osteoporosis therapy during the last decade [16]. As a consequence, a question that arises is whether alterations in the osteocyte biology, here represented by disturbances in the Wnt pathway, could also play a major role in CKD-MBD physiopathology. Activation of this pathway, represented by the intracellular accumulation of β-catenin and its translocation to the nucleus, is linked to an increase in bone formation. Scl, the gene product of SOST, is an inhibitor of Wnt pathway, decreasing bone formation and also increasing resorption [17]. However, the current understanding of Scl synthesis is as yet incomplete. The list of known stimulators of Scl includes calcitriol, bone morphogenic proteins, glucocorticoids, leptin, some cytokines, calcitonin [10] and phosphate [18], whereas mechanical loading and PTH has been shown to inhibit Scl production [16]. As most of CKD patients present elevated PTH, we initially would expect to find low Scl levels in this population. However, Cejka and colleagues have demonstrated elevated Scl in prevalent dialysis patients [7]. In this same direction, an increased bone expression of Scl was found in rodents and patients with CKD [8]. Taken together, these data generated the hypothesis that elevated Scl might partially explain the bone resistance to PTH that is commonly seen in CKD patients. However, it is not clear if circulating Scl reflects only the osteocytic synthesis or could also be a biomarker of vascular calcification, where vascular smooth muscle cells that had undergone phenotypic transition would also secrete this protein attempting to inhibit calcification progress [19]. In line with this theory, Brandenburg and colleagues recently described a positive association between serum Scl levels and aortic valve calcification, as well as an increased Scl expression in aortic valve tissue adjacent to areas of calcification [20]. The recognition of Scl as a biomarker of bone resistance to PTH and of valvular calcification led us to investigate whether this protein could also be associated with adverse outcomes. Indeed, we found that high serum Scl is associated with an increased risk of death in our cohort, decreasing life expectancy in more than five years in a sample of relatively young dialysis patients. This association was independent of age, sex or presence of diabetes mellitus, which are classically linked to higher mortality risk, and of serum FGF 23, potential CKD-MBD markers of mortality.

We are aware that Kaplan-Meier analysis is the most popular method used to estimate time-to-event data, especially time-to-death. However, there is growing evidence in the literature favoring a different approach that taken into account a competing risk event, particularly in nephrology [21, 22]. This is mainly because the dropouts due to kidney transplant might mislead the interpretation of results, as these patients are usually healthier than those patients who are still on dialysis. In our study, due to the long-term follow-up, the number of patients who received transplant was similar to the number of patients who died. Competing risk analysis however, showed that higher Scl, presence of diabetes and age were independently associated to mortality, even when the occurrence of kidney transplant was considered. This statistical method allows us to confirm our results, reducing the possibility of misinterpretation while using the classical Kaplan-Meier.

In the current study, a positive correlation between Scl and calcitonin was found, in accordance with previous experimental study, where the anabolic effect of PTH was blunted by calcitonin [10], implicating this hormone as a negative regulator of bone formation. Curiously, the authors found that calcitonin exerted this effect through the stimulation of Scl synthesis. Since CKD patients present an increase in both circulating Scl [7] and calcitonin [23], an intriguing hypothesis would be that the second could stimulate Scl production, justifying, at least partially, the elevated Scl found in this population. We also found an association of serum Scl and FGF 23, suggesting a mechanistic link that has yet to be determined. Interestingly, we have recently described that predialysis patients who were treated with sevelamer presented simultaneously decreases in both FGF 23 and Scl [24], opening new avenues for additional exploration.

Importantly, the here descript positive association of high serum Scl and increased mortality risk contradicts the previous finding of Viaene and colleagues [9], who have found a protective effect of Scl. However, there are some differences between these studies that could explain these apparent conflicting results. Our patients were approximately 25 years younger, with a lower percentage of diabetes and higher survival rate. Moreover, our analysis was adjusted for the presence of diabetes, the most powerful predictor of survival in our cohort. Finally, this difference could be explained by the different assays that are commonly employed to measure circulating Scl [25]. The fact that our population is demographically different from worldwide ESRD dialysis patients (younger, with low diabetes, and potentially with low cardiovascular risk), does not allow us to impute our results to other populations.

## Conclusions

Our study has important strengths, including its long follow-up and the evaluation of several demographic and also biochemical variables that certainly could influence the outcomes. Also, the competing risk analysis empowered the association between Scl and mortality in a scenario of a large number of kidney transplant competing with the event of interest, death. However, our study also has some limitations that should be considered when interpreting results. First, it has been done in a single center. Second, the population evaluated could not represent the majority of patients that are currently on dialysis worldwide. In addition, association does not mean causation. Third, the long storage period might have interfered in the results. Finally, we used an event per variable ratio that may be considered low for regression [26], which might be associated with overfitting. Nevertheless, considering the high plausibility and the narrow confidence intervals for the association between Scl and the outcome of interest, we do not believe that this could invalidate our findings. Therefore, prospective, interventional studies are needed to elucidate whether clinical interventions to modulate serum Scl would be able to improve these patients survival.

We have demonstrated that high Scl was associated with CV mortality in a prevalent dialysis population, in a competing analysis approach. Further studies are necessary to confirm our data.
